# Ameliorative impacts of propolis against testicular toxicity promoted by doxorubicin

**DOI:** 10.14202/vetworld.2024.421-426

**Published:** 2024-02-20

**Authors:** Khalid M. Alsyaad

**Affiliations:** Department of Biology, College of Science, King Khalid University, Abha 61413, Saudi Arabia

**Keywords:** doxorubicin, histopathology, propolis, testicular toxicity

## Abstract

**Background and Aim:**

Doxorubicin (DOX) is often used as a chemotherapeutic agent, although it may damage testicular functions. This study was designed to investigate the protective effects of propolis on testicular histological changes, semen parameters, and testosterone concentrations as a means of protecting against testicular damage caused by DOX chemotherapy.

**Materials and Methods:**

Forty-eight male Wistar rats were divided into four groups with 12 animals per group. The first group served as the control. Rats in the second group were administered 4 mg/kg DOX. The third group was administered 4 mg/kg of DOX and 30 mg/kg b.w. propolis. The fourth group was orally dosed daily with 30 mg/kg b.w. propolis.

**Results:**

DOX treatment resulted in a significantly decreased weight gain (WG) rate compared with the control, whereas DOX + propolis resulted in improved WG and returned to the normal range. Testosterone levels were comparable among the experimental groups, with a significant increase in the propolis-treated group. In addition, DOX-treated groups exhibited a remarkable depletion in sperm counts, motility, and viability compared to the other groups.

**Conclusion:**

Most of the histological and hormonal changes resulting from the toxicity of DOX returned to almost normal after treatment of rats with the aqueous extract of propolis, indicating that propolis ameliorated the effects of DOX poisoning on testicular function in male rats.

## Introduction

Propolis, also known as bee gum, is a resinous substance placed by bees of different species. It has antioxidant properties as an active medicinal substance because of its phenolic elements, esters, and flavonoids [1, 2]. The overall chemical composition of most propolis is quite similar. According to Anjum *et al*. [3], it is composed of resins (50%), bee wax (30%), essential and aromatic oils (10%), pollen (5%), and other organic components (5%). Esters, flavonoids, terpenes, beta-steroids, aromatic aldehydes, and alcohols are important organic components of propolis [4]. Several studies have been conducted on oxidative inflammation of testicular tissue, which has been shown to have a negative impact on fertilization and sperm production; however, antioxidants may be helpful in reducing or avoiding this damage [5, 6]. Furthermore, propolis acts as an antioxidant and plays an important role in protecting the genitals from toxic exposure and protecting testicular damage caused by the formation of aluminum trichloride, thus preserving fertility [7, 8]. In addition, propolis therapy enhances the weight of organs, body weight, semen characteristics, and testosterone hormone [9]. In addition, biological data demonstrated that all tested propolis-enriched dipping sauce samples improved all cadmium chloride-induced testicular histological and biochemical changes [10]. Doxorubicin (DOX) is a highly effective lipid-coated anticancer drug used in chemotherapy [11–13]. DOX chemotherapy causes oxidative damage and cell death in testicular tissue [14]. DOX damages spermatogonial DNA and sperm morphology, impairs sperm maturation, and reduces epididymal function and semen quantity [15–17].

Thus, the hypothesis for this study was based on the idea that propolis might be an effective way to protect against testicular damage caused by DOX chemotherapy without opposing its pharmacologic action. Therefore, this study aimed to investigate the protective effects of propolis on testicular histological changes, semen parameters, and testosterone levels after DOX-induced testicular chemotherapy.

## Materials and Methods

### Ethical approval

All experiments were reviewed and approved following the rules and regulations of the Research Ethics Committee, King Khalid University (No. ECM#2023-1206).

### Study period and location

The study was conducted from 15 January to 25 February 2023 in the Physiology Laboratory, Department of Biology, College of Science, King Khalid University, Saudi Arabia.

### DOX and propolis extract

DOX and propolis were obtained from Catalogue Sigma-Aldrich-St Company, Louis, Monaco, USA (DOX product No. was D1515 and propolis product No. was 211200). To prepare aqueous propolis extract, 10 g of propolis was mixed for 30 min in 100 mL of boiled distilled water with constant stirring. Subsequently, the extract is stored at 4°C in the dark and in sterile bottles until necessary.

### Animals and experimental protocol

Forty-eight male rats (Wistar, *Rattus norvegicus*), aged 84–98 days and weighing 350–380 g, were divided into four groups (12 animals per group) as follows:

G1: (Control) treated daily with 0.5 mL/injection of physiological saline solution.

G2: DOX was administered at 4 mg/kg/weekly injection into the peritoneal cavity [18].

G3: (DOX + propolis) treated with a weekly injection of 4 mg/kg DOX and a daily oral dose of 30 mg/kg propolis using a stomach tube.

G4: (30 mg/kg) of propolis was orally administered to the animals daily using a stomach tube [8].

Each group was placed in a special plastic cage, and a rectangular piece of white paper was pasted on each cage to differentiate between the groups. The treatment was continued for 42 days.

### Clinical observations and weight of animals

The animals were euthanized at the end of the experiment (after 42 days of the treatment) according to standard procedures and the rules and regulations of the Research Ethics Committee, King Khalid University, No. ECM#2023-1206. The internal organs were checked during autopsies with a dissection board, forceps, scissors, labels for containers, fixatives, and any media or collection tubes or cups that may potentially be needed. Samples were collected for histological analysis. The average weight of all animals in the experimental groups before (WBT) and after (WAT) treatment and weight gain (WG), WG = WBT − WAT were calculated.

### Collection of plasma and estimation of testosterone hormone

In practice, on the 42^nd^ day of the experiment, all the animals were subjected to blood sampling from the retro-orbital plexus. The harvested samples were centrifuged at 3500× *g* for 15 min, and the collected plasma was stored at −20°C until further analysis. Plasma testosterone hormone concentrations were estimated using ready-made kits produced by Dade Behring (Product No: US6153442A), USA, using a Mini VIDAS device (Bimedis Co. USA).

### Semen characterization and sperm

The epididymis and testicular tissues were dissected, separated, and weighed after anaesthetization. One epididymis was examined immediately for sperm viability and abnormalities, while the other was maintained at –80°C for measurement of spermatozoa quantity. The epididymis was divided into sections in two milliliters of tissue culture medium-199 (TCM-199; Sigma-Aldrich Co. St. Louis, MO) sources that had been preheated at 37°C to examine individual motility [19, 20]. A single drop from this mixture was fixed in 95% alcohol and stained in 1% eosin solution for abnormality analysis [21]. Sperm viability, death, and survival were examined using a hemocytometer, as described by Moumen *et al*. [22]. Sperm concentrations were determined according to Kempinas and Lamano-Carvalho [23] with individual epididymis frozen by homogenizer tissue (5 mL 0.9% NaCl + 0.1% thimerosal + 2 drops of 0.1% eosin + 0.1% thimerosal + 0.5% Triton X-100).

### Histopathological analysis

The testicles were removed immediately after slaughter and weighed, and one of the testicles of all males was separated, fixed in Bouin’s for 48 h, and placed in 70% alcohol. Using a computerized tissue system (Sakura, Japan), the samples were handled according to Culling [24], with grades 70%, 80%, 90%, 95%, and 100% of ethanol involved in dehydration, followed by a second application of xylene for cleaning and two modifications of molten paraffin wax for impregnation. We used a microtome (RM2245 model, Leica Biosystems Co., Germany) to slice sequential sections of 4–5 m in size and an embedding machine to block and embed the specimens. An auto-stainer (5020 model, Leica Biosystems Co.), hematoxylin and eosin (H&E), and conventional periodic acid Schiff (PAS) and H&E methods were employed for histochemical staining [25]. A microscope (Olympus DP73, Japan) with a digital camera was used to inspect for changes in the sections.

### Statistical analysis

Biochemical parameters are presented as mean ± standard deviation. A one-way analysis of variance followed by Duncan’s test was used to test for significant differences between means in different groups, and p < 0.05 was considered significant using the Statistical Package for the Social Sciences Version 16.0 (IBM Corp., NY, USA).

## Results

### Clinical observations and mortality rates

No mortality was observed in animals of different groups during the experiment period. In addition, no pathological indicators were found in the internal organs during autopsies, with the exception of mild bleeding spread in the lungs in a number of animals, which could not be attributed to any of the treatment groups because they were found in some organs of the control group. In addition, animals in the DOX group exhibited less physical activity and reduced appetite than those in the DOX + propolis, propolis, and control groups.

### Body weight

Table-1 presents the average weight of all animals in the experimental groups before and after treatment and WG (g). There was a natural improvement in the rate of WG observed in all groups, except for a higher decrease (p = 0.01) in DOX animal weight after treatment and WG compared with the control group. In addition, the propolis group showed a non-significant increase in WG compared with the control group.

**Table-1:** Mean body weight (g) in male rats before and after treatment and WG (g) for all different experimental groups (mean ± standard deviation).

Group (n = 12)	Animal weight before treatment (g)	Animal weight after treatment (g)	WG (g)
Control	363.66 ± 26.72	416.57 ± 18.31	53 ± 4.34
DOX	369.28 ± 32.79	325.55 ± 43.17**	44 ± 3.82**
DOX+propolis	356.11 ± 43.23	395.53 ± 41.57	39 ± 1.13
Propolis	368.18 ± 19.86	428.83 ± 23.54	60 ± 6.46

** Means in the same column differ significantly (p ≤ 0.01). DOX=Doxorubicin, WG=Weight gain

### Weight of testes and epididymis

Table-2 presents the weight of the testes (g) and epididymis (mg) of rats. No difference was observed in the average weight of the testicles and epididymis in all treatment groups except for a significant decrease (p = 0.05) in the DOX group compared to the control group.

**Table-2:** Mean weight of testicles (g) and epididymidis (mg) in male white rats after end of treatment period for all different experimental groups (mean ± standard deviation).

Group (n = 12)	Testes weight (g)	Epididymis weight (mg)
Control	3.28 ± 0.37	337.98 ± 7.63
DOX	2.01 ± 0.13*	197.48 ± 6.83*
DOX+Propolis	2.76 ± 0.37	287.23 ± 4.28
Propolis	3.05 ± 0.22	319.61 ± 7.71

* Means in the same column differ significantly (p ≤ 0.05). DOX=Doxorubicin

### Testosterone hormone

Table-3 shows a significant decrease (p < 0.05) in the testosterone hormone level in the DOX-treated group compared to the control group, and a significant decrease (p < 0.05) was observed in the DOX + propolis group compared to the control group.

**Table-3:** Testosterone concentrations (ng/mL) in male white rats after end of treatment period for all different experimental groups.

Group (n = 12)	Testosterone hormone (ng/mL)
Control	1.416 ± 0.240
DOX	0.664 ± 0.097*
DOX+Propolis	1.033 ± 0.185
Propolis	1.572 ± 0.216

* Means in the same column differ significantly (p ≤ 0.05). DOX=Doxorubicin

### Determination of sperm parameters

Table-4 shows the sperm count (10^6^/mL), viability (%), motility (%), and abnormality forms (%) of sperm in the experimental groups. The results showed that the cauda epididymis stock contained in DOX animals was lower and had a higher decrease (p < 0.01) in the sperm count (×10^6^/mL) compared to the other groups. The data show that animals treated with propolis alone or while receiving chemotherapy from DOX had better (p < 0.05) improvements in viability (%) and motility (%) compared with those in the DOX group. In contrast to the control group, the DOX group experienced a significant decrease (p < 0.05) in these metrics. The percentage of abnormal sperm (%) increased to 43.34% in the DOX group compared with the control group (p < 0.01). Furthermore, the percentage of abnormal forms of sperm decreased significantly (p < 0.01) in the DOX + propolis and propolis groups compared with that in the DOX group.

**Table-4:** Sperm count, viability, motility and abnormality of sperms in male white rats after end of treatment for all different experimental groups.

Group (n = 12)	Sperm count (×10^6^/mL)	Viability (%)	Motility	Abnormal forms (%)
Control	92.21 ± 7.35	82.13	84.41	27.46
DOX	49.71 ± 5.64**	56.36*	55.02*	43.34**
DOX+Propolis	88.54 ± 6.18	60.61	74.68	34.98
Propolis	93.88 ± 5.58	88.36	84.94	23.84

* Means in the same column differ significantly (p≤0.05).

** Means in the same column differ significantly (p ≤ 0.01). DOX=Doxorubicin

### Histological examination

**Figure-1:**
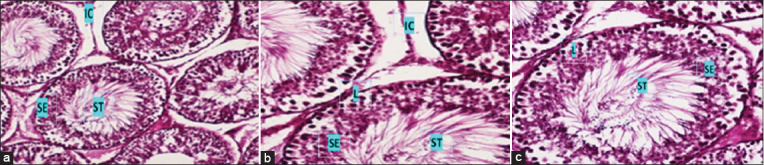
Histological micrographs of the normal testicular tissue of the white rat male from the control group. (a) Shows the regular arrangement of interstitial cells (IC), seminiferous tubules (ST), and lining the seminal tubule with stratified epithelium (SE), which is made up of maternal cells and primary and secondary sperm cells. (b) Shows a magnification of the IC with seminal precursors and filling of the sperm tubule (L) cavity with sperm. (c) Shows the regular arrangement of ST (hematoxylin and eosin [H&E] 200× and H&E 400×).

**Figure-2:**
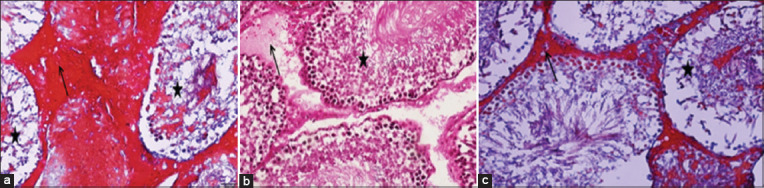
Histological micrographs of the testicular tissue in the male white rat of the doxorubicin-treated group. (a) Shows hemorrhage and focal necrosis and the infiltration of protein substances between the ST (arrows). (b) Shows the dissociation and descent of sperm cells into the ST cavity with vacuolar degenerative changes in the seminiferous tubule tissue (stars) and deformation and zigzag of the basement membrane. (c) Shows the combination of necrosis, infiltration of protein substances, and descent of sperm cells into the sperm tubule cavity.

**Figure-3:**
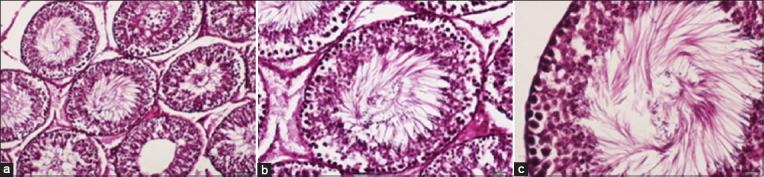
Histological micrographs of the testicular tissue in the male white rat of the doxorubicin + propolis group. (a) Shows the effect of propolis on rearranged structure of testicular tissue represented by the ST, and the interstitial cells in the IC. (b) Shows the regular arrangement of IC. (c) Shows the regular arrangement of ST.

**Figure-4:**
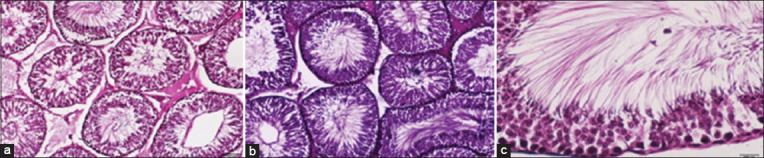
Histological micrographs of the testicular tissue in the male white rat of the propolis group. (a) Shows the regular arrangement of IC, ST, and SE. (b) Shows a marked improvement in the tissue of the ST. (c) Shows a marked improvement associated with a noticeable increase in the number of cells lining them.

Histological imaging confirmed a typical seminiferous tubule and interstitial tissue pattern in the control group. Furthermore, the seminal tubule was lined with stratified epithelium constituted of maternal cells, primary and secondary sperm cells, seminal precursors, and sperm cells, which filled the tubule cavity with sperm cells based on the basement membrane (Figures-1a-c). Histological observations in the DOX group (Figures-2a-c) showed focal necrosis, bleeding, and infiltration of protein substances between the seminiferous tubules. In addition, sperm cells dissociated and descended into the sperm tubule cavity, exhibiting vacuole degenerative changes in the seminiferous tubule tissue as well as deformation and zigzag of the basement membrane. There was a significant enhancement in the tissue structure in animals treated without (Figures-3a-c) and during DOX exposure (Figures-4a-c). Although no pathological changes were observed in the testicle when treated with the aqueous extract of propolis, it had a clear effect on testicular activity compared with the control, with a significant increase in mitotic activity and the number of layers of spermatogonia. Sertoli cells were also observed, and the seminal tubules in the testicle were filled with more mature spermatids than the testicle in the control group animals.

## Discussion

The present study showed that DOX therapy resulted in a significant decrease in average body weight, WG, hunger, and physical activity compared to rats in the control group. Consequently, DOX dramatically decreases the overall antioxidant capability because of its ability to interact with free radicals, which also significantly increases the oxidative pressure [26, 27]. Antioxidant enzymes are significantly inhibited in animals treated with DOX due to the production of numerous free radicals [28].

DOX treatment significantly decreased testosterone levels, testicle weight, and sperm motility in animals compared to the control group. These results are in agreement with those of Yang *et al*. [29], who found that the testicular weight and length were dramatically and gradually lowered as the DOX dosage was sequentially increased.

DOX-treated testicular tissue showed severe bleeding, necrosis, protein infiltration, disintegration of sperm cells, and deformation of seminal tubule. Furthermore, free radicals cause DOX toxicity in rats, resulting in cell death [30]. According to Erbaş *et al*. [31], DOX therapy resulted in programmed cell death, oxidative stress, inflammation, sperm deterioration, and breakdown of testicular tissues.

Compared with the control group, DOX + propolis treatment increased sperm parameters, body weight, and sperm concentration in animals. These results are in agreement with those of Rizk *et al*. [32], who reported that propolis extract may protect the testis from DOX-induced toxicity without reducing its anticancer potential. In addition, propolis has been demonstrated to have a high antioxidant efficacy due to the presence of numerous phenolic compounds, including flavonoids, flavans, hydroxybenzene, hydroxycinnamic acid, and stylbene acids [33].

The administration of propolis in combination with other supplements or alone has also been found to improve organ function [34]. In addition, the group treated with DOX + propolis showed a notable improvement in seminal tubule tissue as well as an increase in the number of cells lining it. In addition, when propolis was applied, the testicular tissue structure was normal and resembled that of the control group. This is a clear example of propolis’s ability to protect against DOX toxicity due to its antioxidant properties. The rationale behind the use of this substance for treating numerous illnesses includes its anti-inflammatory, antibacterial, antiseptic, antifungal, anti-gastrointestinal ulcer, anticancer, tonic properties, and immunomodulatory properties [35–37].

These results indicate that propolis alters the concentration of testicular enzymes and has a protective effect against testicular injury. Propolis’s protective role stems from its antioxidative capacity, with flavines acting as free radical scavengers that protect against oxidative pressure and metabolic disorders [38]. Free radicals are continuously and permanently formed in the body from enzymatic and non-enzymatic reactions in tissues [39]. They perform physiological functions, such as information transfer and communication, and there is a natural balance between food and body production; however, increased free radicals can cause damage to cells and tissues [40].

## Conclusions

This study demonstrated the preventive effect of aqueous propolis extract. The toxic effects of DOX on the testicles of rats have also been reduced. In addition to improving semen parameters and helping to increase the caudal epididymis stock of sperm and supporting their activity, vitality, and increasing the percentage of individual motility.

## Author’s Contributions

KMA: Conceptualization, methodology, data analysis, validation, writing-original draft and editing. The author has read, reviewed, and approved the final manuscript.
